# Soil respiration from fields under three crop rotation treatments and three straw retention treatments

**DOI:** 10.1371/journal.pone.0219253

**Published:** 2019-09-23

**Authors:** Dejie Kong, Nana Liu, Weiyu Wang, Kashif Akhtar, Na Li, Guangxin Ren, Yongzhong Feng, Gaihe Yang

**Affiliations:** 1 College of Agronomy, Northwest A & F University, Yangling, Shaanxi, China; 2 Agricultural Biotechnology Center of NingXia Academy of Agriculture and Forestry Sciences, Ningxia Yinchuan, China; 3 Research Center for Recycling Agriculture Engineering Technology of Shaanxi Province, Yangling, Shaanxi, China; 4 Ningxia Agricultural Institute of Survey Design, Yinchuan, China; Tennessee State University, UNITED STATES

## Abstract

Straw retention is an effective method to conserve soil water content and improve soil carbon stocks. However, how soil carbon dynamics respond to different straw retention practices remains unclear. In this study, we investigated soil respiration and soil carbon sequestration at depths of 0–100 cm. We conducted a two-year field experiment with three crop rotation treatments and three straw retention treatments in northwest China. The straw retention treatments included no straw retention (NS), retention of half the straw (HS), and retention of the total amount of straw (TS). The crop rotations treatments included winter wheat plus summer soybean (WS), winter wheat plus summer maize (WM), and winter wheat plus summer fallow (WF). Mean soil respiration rates under WS, WM, and WF treatments were 5.14, 6.53, and 5.49 μmol·m^-2^·s^-1^; and 5.67, 5.47, and 6.03 μmol·m^-2^·s^-1^ under TS, HS, and NS treatments. The mean soil water content were 15.50%, 15.57%, and 15.74% under WS, WM, and WF rotations, and 15.81%, 15.41%, and 15.50% under TS, HS, and NS treatments. The soil organic carbon (SOC) concentration was higher with increased straw retention, and lower at deeper soil depths. Mean SOC concentrations under different rotations and straw treatments of TS, HS, and NS, respectively were as follows: WS: 6.91, 6.63, 6.39 g/kg; WM: 6.90, 6.72, 6.57 g/kg; and WF: 6.49, 6.52, 6.37 g/kg. Soil temperature was the main determinant of soil respiration rates. We conclude that WS rotation resulted in lower soil respiration, WM rotation resulted in a higher soil carbon sequestration potential, and WF rotation resulted in higher soil water content. However, continued, long-term monitoring is needed to confirm the effect of rotations and straw retention on soil respiration and carbon sequestration in dryland cropping systems in northern China.

## Introduction

Agricultural soil has been considered one of the largest emitters of greenhouse gas (GHG) emissions globally [1]. Currently, agriculture-related activities, and land use change in particular, contribute a significant portion (approximately 20%) of global GHG emissions [2]. Agriculture is one of the top GHG emitters in China, with an estimated total emission of 686 Mt CO_2_ equivalent (CO_2_-e) in 2007, representing 9.2% of the nation’s total [3]. Soil respiration is the exchange process of carbon dioxide (CO_2_) between the soil and atmosphere, and generally includes four biotic process, namely plant root respiration, soil microbial respiration, soil animal respiration, and soil organic matter decomposition [4]. As such, soil respiration is generally related to the soil–atmosphere CO_2_ concentration gradient, and the atmospheric CO_2_ concentration, microbial and enzyme activities, and physicochemical properties of the soil surface may affect soil respiration rates [1, 5]. Large natural fluxes of soil respiration are likely to increase due to changes in the Earth's condition [6]. Tillage [7], N fertilization [8], organic fertilization [9] and soil temperature and soil water content have been identified as the most important environmental factors influencing soil respiration [10–11]. However, few studies have focused on soil respiration under three rotation modes and three straw retention amounts.

Research on straw retention has a long tradition, and China is a large agricultural country with the highest straw yield in the world [12]. However, most of the straw in China is used for cooking and heating in rural areas [13, 14], and a large amount of crop residues is regularly removed [15] or commonly burned in fields following harvest for reduce labor, contributing to China^’^s serious air pollution problems in recent years [16]. As such, agricultural areas in China mainly depend on fertilizer inputs to maintain crop yields [17]. Previous studies revealed that the rational utilization and sustained application of straw retention is important for sustainable agriculture, including improving soil fertility and soil organic carbon (SOC) concentrations [18]. SOC plays a vital role in determining soil fertility, water holding capacity, and susceptibility to land degradation [17], and it is widely used to assess the environmental performance of cropping systems, since it is strongly influenced by climate, soil physico–chemical characteristics, land management, and carbon (C) input from crop retention [19]. However, it has been widely reported that straw retention can increase soil respiration [20], although some studies have also demonstrated that straw retention can reduce soil respiration [21], and the overall trends and magnitude of changes in soil C in response to straw return remain uncertain [20]. Therefore, it is important to quantitatively evaluate the effects of straw retention on soil respiration and soil organic carbon concentrations.

Crop rotations and straw harvest are performed worldwide [22], and crop rotation is an ancient agricultural practice that has been used for thousands of years in China. Summer soybeans and winter wheat, summer maize and winter-wheat, and summer fallow and winter wheat have comprised the main rotation modes of planting systems in western China for thousands of years. Previous studies demonstrated that properly designed and managed rotation systems can overcome the based consumption of soil, improve agricultural productivity and, accordingly, keep agriculture sustainable [23]. Crop rotation can reduce the amounts of agro-chemicals needed, and improve conditions for soil organisms, improve soil texture and structure, improve root penetration and water availability, improve soil fertility, and increase yields [22]. Crop rotation has been retained in modern agriculture throughout the world. However, the practice of crop rotation by Chinese farmers in traditional farming systems has been gradually abandoned [24], despite the fact that crop rotation can maintain high system productivity, result in economic benefits, improve energy productivity, reduce the carbon footprint, and increase energy use efficiency [25]. As such, it is essential to improve cropping system management to mitigate greenhouse gas (GHG) emissions. However, there is a lack of studies that evaluate the effects of cropping systems on soil respiration and changes in soil organic carbon (SOC) concentrations using long-term experiments with winter wheat and summer crops such as Leguminosae (C3), Gramineae (C4), and fallow rotation modes.

Dryland areas cover approximately 41% of the Earth’s surface, and sustain over 2 billion inhabitants [26]. The soil water supply is the main factor that limits dryland crop production in China. Arid and semi-arid regions account for half of the land area in China [27]. In the typical rain-fed farming areas of this region, the shortage of water is very serious, and it is important to enhance the crop utilization efficiency of soil water [28]. The soil water content is often too low to allow rapid seed germination [29]. Soil wetting depth increased with increases in mulch rates, and straw mulching has the potential to increase soil water storage from small amounts of precipitation [30]. Knowledge of soil respiration and the influencing factors (such as land-cover types [4], and biotic and abiotic processes [31]) is essential to further understand responses of carbon dynamics in soils to climate change. It is widely known that crop rotations and crop retention do fulfill important agronomic functions of sustainable development of agriculture [22]. However, our currently poor understanding of soil respiration is one of the major sources of the uncertainty in estimates of soil carbon dynamics under different rotation and straw retention systems in arid regions.

Soil respiration and SOC concentrations could be affected by different straw retention and rotation treatments. Therefore, field experiments were conducted in the dryland farming village of Yangling. The objectives of this study were to examine the effects of straw retention combined with different rotation treatments on soil respiration, soil water content dynamics, and the responses of soil respiration to soil temperature and soil water content. We further aimed to provide a theoretical basis for the dryland farming soil carbon cycle process, and aid policy makers in making decisions for the sustainable development of modern agriculture.

## Materials and methods

### Study site

**Fig 1:**
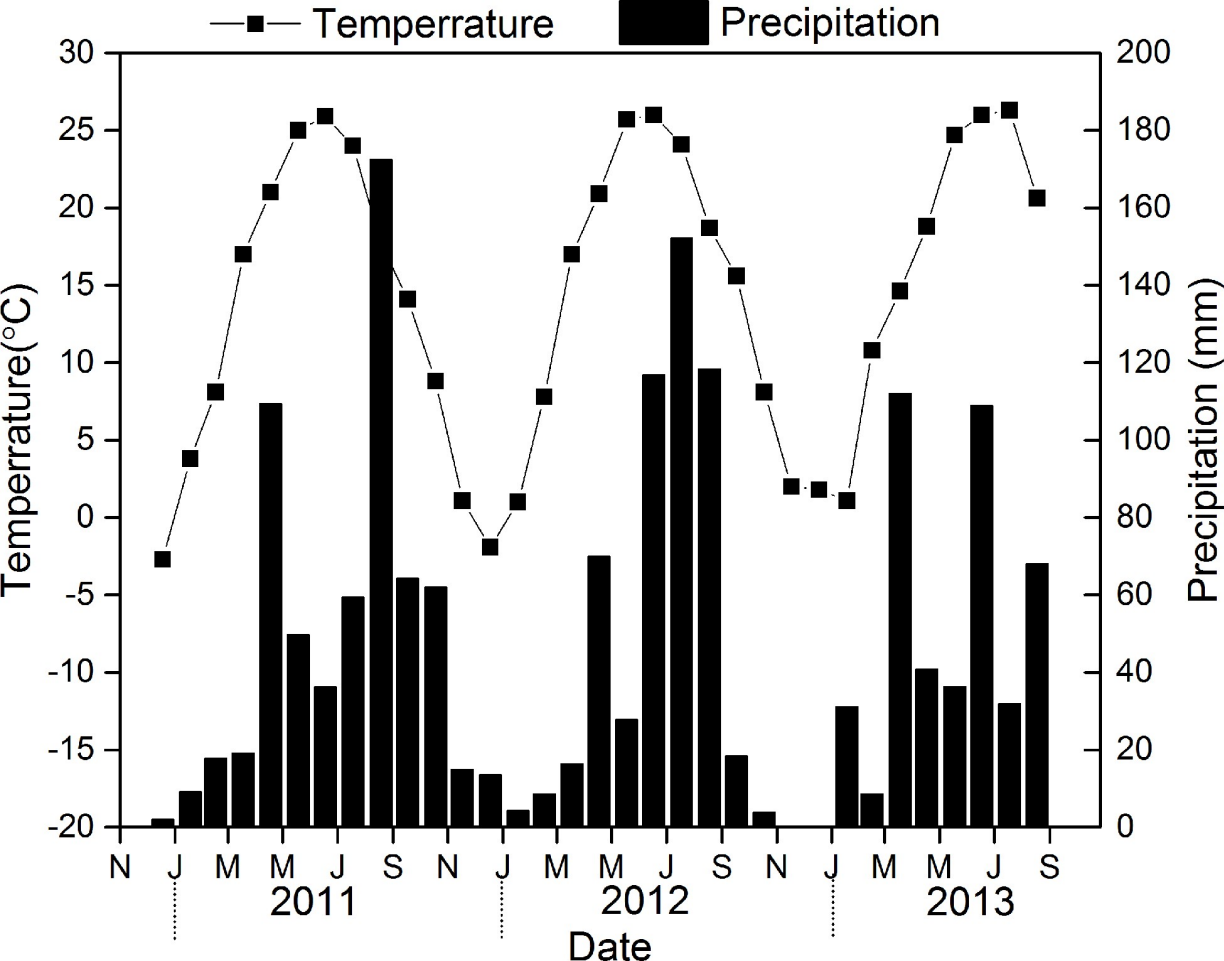
Mean monthly temperature and total precipitation during the crop growing seasons in the study area from January 2011 to June 2013.

This experiment was conducted at the Northwest A & F University campus experimental field, which is located at 520 meters above sea level in Yangling village, Ghuanzhong region, Shaanxi Province, north-west China (34°12′N and 108°7′E; the study area is open public domain and not protected in any way, and the field studies do not involve endangered or protected species). This area has a typical warm, temperate, semi-humid monsoon climate. The mean annual rainfall is 630 mm, and the precipitation is uneven, mainly falling from July to September, which are also the warmest months, with a mean monthly temperature of 23.4°C and a relative humidity of 72% (Fig 1). The annual average temperature is 12.9°C, and the coldest months are from December to January, with a mean monthly temperature of 0.4°C, and a relative humidity of 67% (Fig 1). The soil (0–20 cm) at the experimental site is Lou soil, and the texture is silt clay loam. The saturated soil water content is 42.8%, the field capacity is 23%, and the bulk density is 1.49 g cm^-3^. In 2008, the soil was found to contain 12.74 mg kg^-1^ alkali hydrolyzable nitrogen, 154.52 mg kg^-1^ available potassium, 21.72 mg kg^-1^ available phosphorus, and 8.57 g kg^-1^ organic matter.

### Experimental design and management

**Fig 2:**
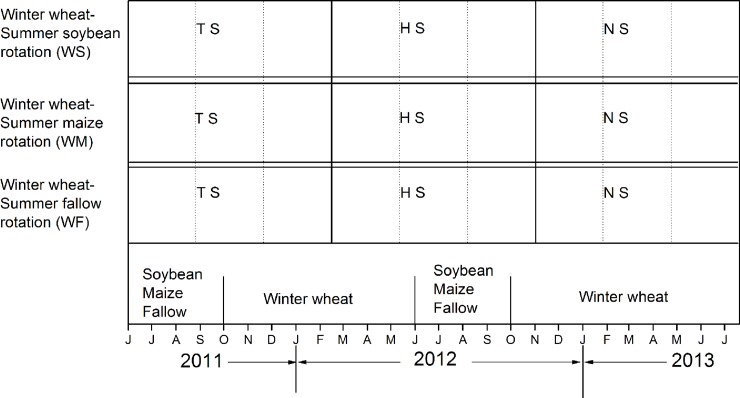
Experiment design and arrangement of crop planting time. TS, total amount straw retention; HS, Half amount straw retention; NS, No straw retention; WS, winter wheat plus summer soybean rotation; WM, winter wheat plus summer maize; WF, winter wheat plus summer fallow. The follow is as some.

The study was conducted in an on-going field experiment that was established in 2008. The field experiments were designed in split-plots, with rotation modes as main plots and straw retention treatments as subplots, with three replications of each treatment (Fig 2). Three rotation modes have been adopted in the study area, and included a winter wheat (*Triticum aestivum* L.) and summer soybean (*Glycine max* (Linn.) Merr.) rotation (WS), a winter-wheat and summer maize (*Zea mays* L.) rotation (WM), and a winter-wheat and summer fallow (WF) rotation. Three straw retention treatments were included. The first was the retention of the total amount of straw (TS). During the winter wheat harvest, the straw mulching treatments were maintained for the next summer soybean or maize crop, and when maize or soybeans were harvested, the straw was returned to the soil before winter wheat sowing. A straw chopping machine was used to smash the straw stalks to obtain even coverage. The second straw retention treatment included retaining half the straw (HS). This involved removing the straw, and then, as in the TS treatment, the straw was chopped to provide even coverage, corresponding to half the amount of the TS coverage. The third straw retention treatment involved no straw retention (NS). In this treatment, all of the straw stalks were removed (Fig 2). Fertilizer was applied before sowing, and was based on local practices. Fertilizing practices consisted of applying 300 kg of phosphorus pentoxide (P_2_O_5_) ha^-1^ and 300 kg of urea (CON_2_H_4_) ha^-1^ as base fertilizer once during the winter wheat season. A further 300 kg of urea (CON_2_H_4_) ha^-1^ was applied as topdressing fertilizer during the summer maize jointing stage of the WM rotation system.

Winter-wheat (xinong889, is a major local winter-wheat varieties selected by the experiment, row spacing 20 cm) was harvested, and the straw was left in the field. Summer soybean (dongdou 339, major local soybean varieties, plant density 15 cm × 60 cm), and maize (Luodan 9, local main varieties, plant density 25 cm × 60 cm) was sown using a seed drill machine at depths of 3–5 cm. We used zero tillage to limit the soil disturbance, but to ensure germination we used rotary tillage at 0–10 cm of soil depth [32]. Each split plot covered a surface area of 12 × 5.1 m^2^, and was separated from other plots by a 0.3 m buffer strip. All of the treatment management measures were in accordance with local conventional cultivation practices. No irrigation was provided beyond rainfall during wheat management.

### Sampling and measurements

#### Measurement of CO_2_ fluxes

Soil CO_2_ fluxes were measured using the open-flow dynamic method with an infrared gas analyzer [33, 34]. All measurements were conducted between 9:00 and 11:00 AM based on the optimal time to represent the average daily CO_2_ efflux in this region [9, 11, 35]. In the case of rain, measurements were conducted 1–2 days after its cessation. The measurement of soil CO_2_ flux was similar to that described by Mariko et al. [33]. The system was composed of one reference line and four identical sample lines connected to a portable infrared CO_2_ analyzer with a data logger (Model GXH-3010E1, Huayun Co., Ltd., Beijing, China [5]). Each chamber had two parts; the lower part was a chamber made of PVC with two ports for the inlet and outlet of air. This chamber was 15 cm in height and 16 cm in diameter, and the bottom edge of the chamber was pushed 4 cm into the soil and positioned in the center of each plot. Live or standing dead vegetation was carefully avoided. During measurements, the ambient air entered the chambers at a flow rate of 0.81L^.^ min^-1^, and airflow to the infrared CO_2_ analyzer was maintained at 0.5 L^.^ min^-1^ below the chamber flow through rate. The upper part, a 2.5 cm high PVC lid, was placed on the top of the body immediately before measurement. In this system, a complete measurement of one sample was obtained within 3 min.

We calculated the soil respiration rate (μmol·m^-2^·s^-1^) as the difference in CO_2_ concentration in the air in the chambers at the beginning and during the measuring time, as follows [9]:

$$F=k\left(X_{2}‐X_{1}\right)H/△t\cdot M$$ Eq 1

Where:

F: CO_2_ respiration rate of soil (μmol·m^-2^·s^-1^)

k: a reduction coefficient, 0.56

X_1_: the mass fraction of CO_2_ at the beginning of the measurement (mg^-1^·kg^-1^)

X2: the mass fraction of CO_2_ at the moment of the measurement (mg^-1^·kg^-1^)

Δt: measuring time (s)

M: the molar mass of CO_2_, 44.01.

### Measurement of soil water content, soil organic carbon, and soil temperature

In addition to CO_2_ fluxes, field soil temperature and soil water content were measured near the field chamber. We measured soil temperatures at depths of 5, 10, 15, 20 and 25 cm using a geo-thermometer buried in the middle of the crop rows after seeding.

During crop growth, soil samples from depths of 0 to 100 cm were collected using a soil auger. Three cores were collected in each plot, and measured at 10 cm depth intervals. The soil samples were divided into two parts, one for soil water content test and the other for soil organic carbon test. Every sample was transported to the laboratory using a closed aluminum box. The oven-drying method was adopted for measuring soil water content, and soil water content was measured by drying samples at 105°C for 6–8 h. The other samples were air dried at room temperature after removing coarse roots and easily detectable crop residues, and ground to pass through a 2-mm sieve [18]. Soil organic carbon (SOC) concentrations were determined using the K_2_Cr_2_O_7_ oxidation method [1].

### Statistical analyses

All data were investigated using mixed-model analysis with SPSS software (SPSS Institute Inc., 2008). Homogeneity of variance and normality tests were conducted on the data (accepted significance level: P = 0.05). When an F-test indicated statistical significance at P < 0.01 or P < 0.05, Duncan's new multiple range method was used to separate the means of the main effect and the significant interaction effects, and to compare the average soil respiration rate of each treatment directly. Correlations between soil water content, soil temperature, and SOC, as well as soil respiration components were determined using redundancy analysis (RDA). We also used analysis of similarities (ANOSIM) to determine the significance of separation across rotation and straw retention treatments [36].

## Results

### Soil respiration

**Fig 3:**
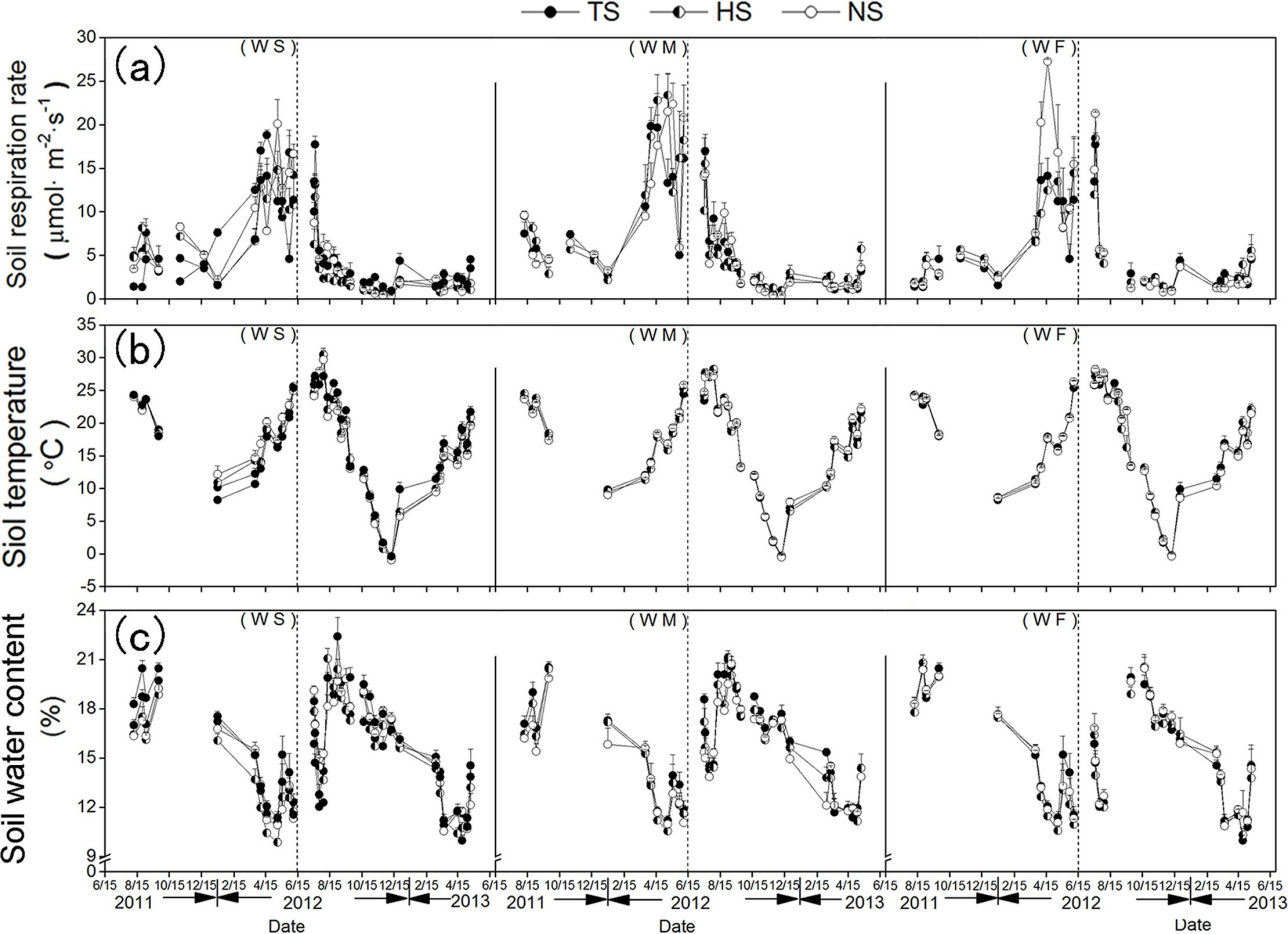
**Changes of soil respiration rate (a), soil temperature (b) and soil water content(c) from the field of treatments under different crop rotation and straw retention amounts**. Bars show means± s. e. m. The follow is as some.

The soil respiration rates under the three rotation treatments and three straw retention treatments followed the same trend (Fig 3A). The results indicate that the lowest rates of soil respiration occurred in the coldest soil, and the highest soil respiration rates occurred in the soil with the highest temperature. The mean soil respiration rates under the WS, WZ, and WF rotation treatments were 5.14, 6.53, and 5.49 μmol·m^-2^·s^-1^, respectively. The mean soil respiration rates were 5.66, 5.47, and 6.03 μmol·m^-2^·s^-1^ under the TS, HS, and NS treatments respectively.

**Fig 4:**
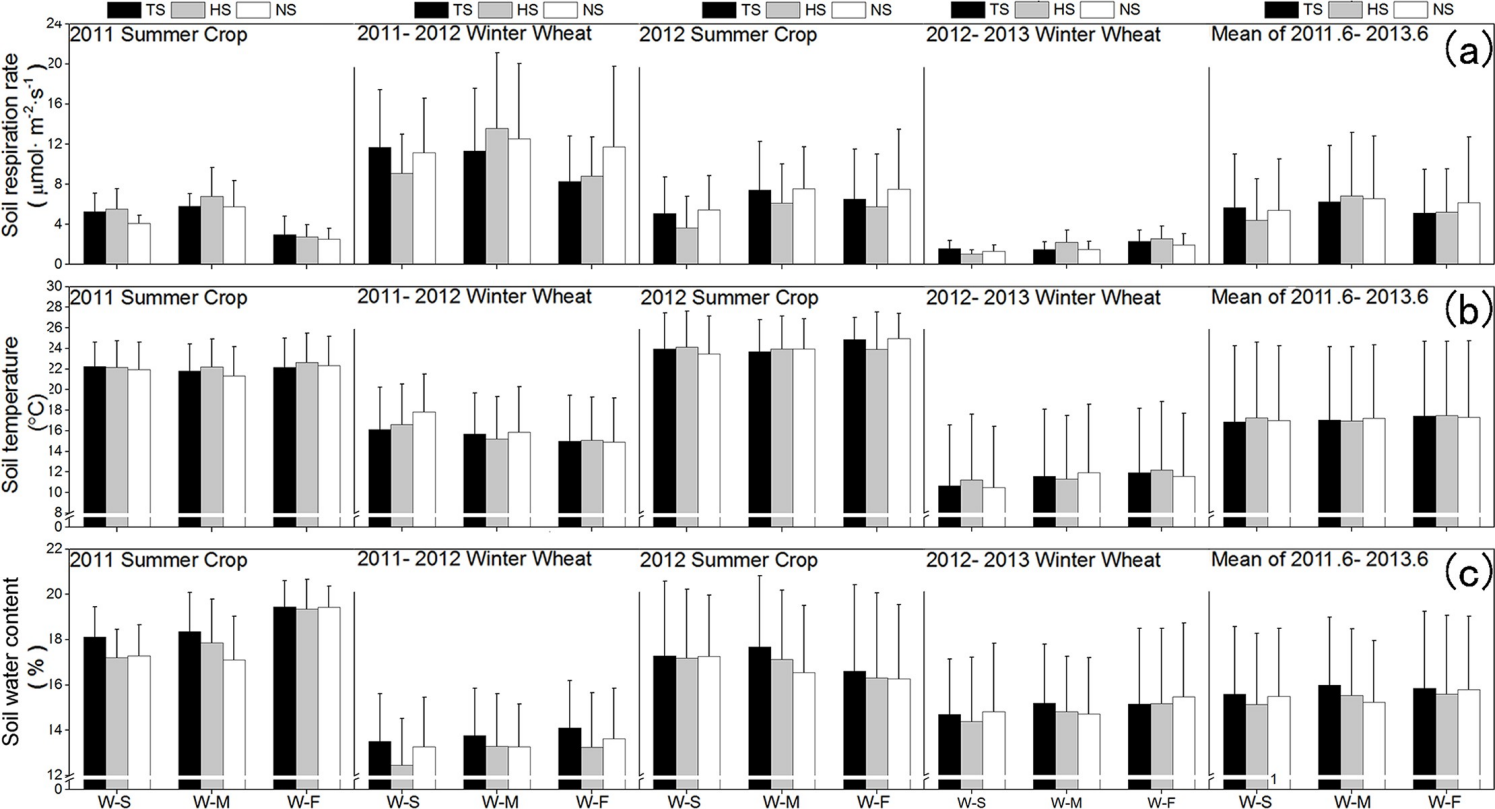
Mean soil respiration rate (a), soil temperature (b) and soil water content(c) in different crops growth period of different treatments.

During the 2011 summer crop growing season (Fig 4A), out of the four monitoring missions that were conducted, we found highly significant differences twice in the soil respiration test among the three rotation treatments (Table 1), and only once among the three straw retention treatments. The average soil respiration rate of the straw retention TS and HS treatments under the three rotation treatments was higher than in soils without straw retention (NS). The mean soil respiration rates under the WS, WZ, and WF rotation treatments during the 2011 summer growth period were 4.97, 6.12, and 2.75 μmol·m^-2^·s^-1^, respectively, and the mean soil respiration rates under the TS, HS, and NS treatments were 4.68, 5.03 and 4.13 μmol·m^-2^·s^-1^ respectively. During the 2012 summer growth period, out of the ten monitoring missions that were conducted, highly significant and significant differences in the soil respiration test between the three rotation treatments were found one and four times, respectively (Table 1), and only once were significant differences in the soil respiration test between the three straw retention treatments found. The mean soil respiration rates under the WS, WZ, and WF rotation treatments were 4.72, 7.03, and 6.59 μmol·m^-2^·s^-1^, respectively, and the mean soil respiration rates were 6.34, 5.16 and 6.83 μmol·m^-2^·s^-1^ under the TS, HS, and NS treatments, respectively.

**Table 1:** Effect of crop rotation and straw retention mounts on soil respiration, soil temperature and water content during the period from 2011 to 2013. (**P<0.01).

Time	Source	Soil Respiration (μmol·m^-2^·s^-1^)		Soil Temperature (°C)		Water Content (%)	
		F Value	Significance	F Value	Significance	F Value	Significance
**2011 Summer crop**							
2011/8/9	rotation	47.505	0.002 **	0.817	0.444	5.916	0.004 **
	straw	0.509	0.636	3.032	0.052	0.962	0.386
2011/8/25	rotation	32.169	0.003 **	33.555	0.000 **	15.462	0.000 **
	straw	7.184	0.047 *	3.236	0.043 *	0.740	0.480
2011/9/1	rotation	1.790	0.190	3.583		36.991	0.000 **
	straw	2.309	0.123	1.909	0.152	0.931	0.398
2011/9/25	rotation	0.521	0.601	7.190	0.001 **	4.604	0.012 *
	straw	1.265	0.302	5.340	0.006 **	1.163	0.317
**2011–2012 Winter wheat**							
2011/11/5	rotation	0.320	0.743	-	-	-	-
	straw	0.622	0.582	-	-	-	-
2011/12/20	rotation	2.194	0.227	-	-	-	-
	straw	1.914	0.261	-	-	-	-
2012/1/15	rotation	0.471	0.655	-	-	-	-
	straw	0.618	0.584	-	-	-	-
2012/3/26	rotation	2.329	0.121	10.142	0.000 **	0.001	0.999
	straw	0.416	0.665	0.480	0.620	1.468	0.236
2012/4/6	rotation	0.366	0.715	14.074	0.000 **	2.381	0.098
	straw	0.383	0.704	1.361	0.260	1.730	0.183
2012/4/18	rotation	0.852	0.492	0.055	0.000 **	2.564	0.082
	straw	0.078	0.927	4.121	0.018	3.663	0.029*
2012/5/8	rotation	2.512	0.104	10.600	0.000 **	0.001	
	straw	3.243	0.058	1.195	0.306	7.802	0.001 **
2012/5/17	rotation	6.823	0.049 *	7.165	0.001**	1.554	0.217
	straw	2.336	0.209	3.272	0.041*	9.831	0.000 **
2012/5/30	rotation	1.029	0.436	9.778	0.000 **	0.364	0.696
	straw	0.337	0.733	2.976	0.055	0.907	0.407
2012/6/7	rotation	3.055	0.068	7.532	0.001 **	0.125	0.882
	straw	2.025	0.157	0.270	0.763	2.408	0.096
**2012 Summer crop**							
2012/7/16	rotation	4.346	0.026 *	1.951	0.146	1.854	0.162
	straw	1.863	0.179	1.160	0.317	0.784	0.459
2012/7/18	rotation	12.338	0.019 *	13.024	0.000 **	8.251	0.000 **
	straw	0.401	0.694	0.485	0.617	1.116	0.332
2012/7/26	rotation	1.767	0.194	10.085	0.000 **	14.874	0.000 **
	straw	0.880	0.429	0.744	0.477	0.716	0.492
2012/8/3	rotation	6.783	0.052	8.375	0.000 **	15.771	0.000 **
	straw	1.934	0.258	4.255	0.016 *	0.963	0.385
2012/8/11	rotation	5.730	0.031 *	15.329	0.000 **	40.733	0.000 **
	straw	3.970	0.043 *	0.151	0.860	3.645	0.030 *
2012/8/23	rotation	5.796	0.138	12.665	0.000 **	1.817	0.183
	straw	4.048	0.198	0.795	0.454	2.122	0.128
2012/8/30	rotation	5.546	0.034 *	6.340	0.002 **	0.023	0.881
	straw	0.773	0.480	2.195	0.116	0.870	0.424
2012/9/6	rotation	8.677	0.099	0.000	1.000	0.271	0.605
	straw	2.510	0.285	0.011	0.989	3.492	0.037 *
2012/9/15	rotation	9.674	0.008 **	11.968	0.000 **	10.873	0.002 **
	straw	0.794	0.473	1.304	0.275	1.193	0.310
2012/9/23	rotation	0.338	0.732	0.000		3.701	0.059
	straw	0.310	0.750	5.758		2.034	0.140
**2012–2013 Winter wheat**							
2012/10/19	rotation	8.907	0.001 **	1.007	0.368	23.226	0.000 **
	straw	0.126	0.882	2.258	0.109	3.884	0.024 *
2012/10/30	rotation	1.705	0.291	9.434	0.000 **	14.852	0.000 **
	straw	1.894	0.264	1.282	0.281	1.632	0.201
2012/11/9	rotation	24.170	0.000**	0.129	0.879	25.434	0.000 **
	straw	1.305	0.291	0.555	0.576	0.848	0.431
2012/11/24	rotation	4.027	0.110	3.339	0.039 *	8.619	0.000 **
	straw	3.576	0.129	0.383	0.683	1.965	0.146
2012/12/10	rotation	3.191	0.148	2.169	0.119	2.374	0.099
	straw	0.980	0.450	0.083	0.920	1.437	0.243
2012/12/26	rotation	7.513	0.003 **	0.001		0.104	0.901
	straw	0.370	0.695	0.000	1.000	1.730	0.183
2013/3/5	rotation	1.449	0.262	22.749	0.000 **	0.267	0.766
	straw	0.304	0.742	0.177	0.838	0.000	
2013/3/13	rotation	1.859	0.269	3.475	0.035 *	2.986	0.055
	straw	1.211	0.388	0.073	0.930	3.110	0.049 *
2013/3/20	rotation	2.148	0.232	4.461	0.014 *	1.615	
	straw	1.696	0.293	0.019	0.981	2.353	0.101
2013/4/15	rotation	0.371	0.711	4.642	0.011 *	4.076	0.020 *
	straw	0.155	0.861	0.198	0.821	0.842	0.434
2013/4/24	rotation	5.538	0.070	4.699	0.011 *	3.129	0.048 *
	straw	1.613	0.306	0.159	0.853	2.871	0.062
2013/5/3	rotation	1.365	0.276	3.561	0.032	12.136	0.000 **
	straw	0.871	0.432	1.045	0.355	0.359	0.700
2013/5/10	rotation	3.770	0.133	7.548	0.001 **	3.174	0.046 *
	straw	0.359	0.721	1.290	0.279	1.885	0.158

During the winter wheat growing season of 2011–2012, out of the 10 monitoring missions that were conducted, significant differences in the soil respiration test between the three rotation treatments were found only once (Table 1), and there were no significant differences in the soil respiration test between the three straw retention treatments. The mean soil respiration rates under the WS, WZ, and WF rotation treatments in 2011–2012 were 10.64, 12.48, and 9.60 μmol·m^-2^·s^-1^, the mean soil respiration rates under the TS, HS and NS treatments in 2011–2012 were 13.79, 13.00, and 13.39 μmol·m^-2^·s^-1^, respectively. During the winter wheat growing season of 2012–2013, out of the 13 monitoring missions that were conducted, we found highly significant differences in the soil respiration test between the three rotation treatments three times (Table 1), and there were no significant differences in the soil respiration test between the three straw retention treatments. The mean soil respiration rates under the WS, WZ, and WF rotation treatments in 2012–2013 were 1.29, 1.70, and 2.25 μmol·m^-2^·s^-1^, and the mean soil respiration rates under the TS, HS, and NS treatments were 1.77, 1.91, and 1.55 μmol·m^-2^·s^-1^, respectively.

### Soil temperature

The variation trends of soil temperature under different treatments during the same growth period were similar. Soil temperature varies seasonally, mainly due to variations in air temperature and precipitation. The results indicate that the trends in soil temperature were similar to the trend of the monthly average temperature during the whole study period. Fig 3B shows the changes in mean soil temperature at the 0–25 cm layer of soils under different straw retention and rotation treatments. Fig 4B shows the mean soil temperature under the different period. During the same period, there were only slight differences in the soil temperatures under different treatments. We found highly significant and significant differences in the soil temperature test between the three rotation treatments 19 and five times, and between the three straw retention treatments were one and three times, respectively (Table 1). The mean soil temperature under WS, WZ, and WF rotation treatments were 17.02, 17.06, and 17.37°C, and were 17.10, 17.20, and 17.16°C under the TS, HS, and NS treatments during two years, respectively.

During the 2011 summer crop growing season (Fig 4B), out of the four monitoring missions that were conducted, we found highly significant differences in the soil temperature test twice between the three rotation treatments (Table 1), and highly significant once and significant differences once, respectively, in the soil temperatures test between the three straw retention treatments. The mean soil temperatures during the summer harvest in 2011 were 22.12, 21.78, and 22.34°C under the WS, WZ, and WF rotation treatments, respectively. The mean soil temperatures in 2011 were 22.08, 22.32, and 21.88°C for soils under the straw retention treatments of TS, HS, and NS, respectively. During the 2012 summer crop growing season (Fig 4A), out of the 10 monitoring missions that were conducted, we found highly significant differences in the soil temperature test between the three rotation treatments seven times (Table 1), and significant differences in the soil respiration test between the three straw retention treatments once. The mean soil temperatures in 2012 were 23.85, 23.87, and 24.57°C for soils under the WS, WZ, and WF rotation treatments, respectively. The mean soil temperatures in 2012 were 24.17, 24.00, and 24.12°C for soils under the straw retention treatments of TS, HS, and NS, respectively.

During the winter wheat growing season of 2011–2012, out of the seven monitoring missions that were conducted, we found highly significant differences in the soil temperature test between the three rotation treatments every time (Table 1), and there were significant differences in the soil respiration test between the three straw retention treatments once. The mean soil temperatures (0–25 cm) during the winter wheat cultivation in 2011–2012 were 16.86, 15.59, and 14.98°C under the WS, WZ, and WF rotation treatments, respectively. The mean soil temperatures during the winter wheat cultivation in 2011–2012 were 15.61, 15.63, and 16.20°C for soils under the straw retention treatments of TS, HS, and NS, respectively. During the winter wheat growing season of 2012–2013, out of the 13 monitoring missions that were conducted, we found highly significant and significant differences in the soil temperature test between the three rotation treatments three and five times, respectively (both P > 0.05, Table 1), and there were no significant differences between the three straw retention treatments (Table 1). The mean soil temperatures (0–25 cm) in 2012–2013 were 10.77, 11.58, and 11.87°C under the WS, WZ, and WF rotation treatments, respectively. The mean soil temperatures in 2012–2013 were 11.37, 11.53, and 11.31°C for soils under the straw retention treatments of TS, HS, and NS, respectively.

### Soil water content

The soil water content dynamics of the nine treatments exhibited the same trend. The soil water content was low during the winter wheat growth stage from March to June, adequate during the summer crop growth stage from August to October month, and remained stable during the other periods, and decreased with increasing soil depth (Fig 3C). We found that the mean soil water content under the WF rotation treatment was higher than that of soils under the WS and WM rotation treatments under the same rainfall conditions over two years. Results revealed that the mean soil water content under the WS, WZ, and WF treatments were 15.40%, 15.57%, and 15.74% respectively. The mean soil water content from 2011 to 2013 (based on 35 measurements taken at 0–100 cm depth) under the TS, HS, and NS treatments were 15.81%, 15.41%, and 15.50%, respectively.

During the two-year summer crop growth period, the soil water content was higher when the precipitation increased. The percentage of soil water content was approximately 17–21% (Fig 4C), corresponding to the highest soil water content during the whole year. During the 2011 summer crop growing season, out of the four monitoring missions that were conducted, we found highly significant and significant differences in the soil water content test between the three rotation treatments three and one times, respectively (Table 1), and there were no significant differences between the three straw retention treatments. The mean soil water content in 2011 were 17.54%, 17.77%, and 19.41% under the WS, WM, and WF rotation treatments, respectively. The mean soil water content in 2011 were 18.64%, 18.14%, and 17.94% under the straw retention treatments TS, HS, and NS, respectively. During the 2012 summer crop growing season, out of the 10 monitoring missions that were conducted, we found highly significant differences in the soil water content test between the three rotation treatments five times, and significant differences between the three straw retention treatments twice, respectively. The mean soil water content in 2012 were 17.25%, 17.13%, and 16.40% under the WS, WM, and WF rotation treatments, respectively. The mean soil water content in 2012 were 17.20%, 16.88%, and 16.70% under the straw retention treatments TS, HS, and NS, respectively.

During the winter wheat growth period, the soil water content was approximately 8% to 15% (Figs 3C and 4C). From the jointing and booting stage to the maturity stage of winter-wheat, the soil water content deceased sharply due to increases in the leaf area, evapotranspiration intensity, and temperature. Evaporation rates were slightly higher for this period than the others in the winter wheat growth stage. In addition, there was little precipitation during this period, resulting in the lowest soil water content during the wheat growth period. During the winter wheat growing season of 2011–2012, out of the seven monitoring missions that were conducted, we found highly significant and significant differences in the soil water content test between the three straw retention treatments twice and once, respectively (Table 1), and there were no significant differences in the soil water content test between the three rotation treatments. The mean soil water content in 2011–2012 were 13.08%, 13.44%, and 13.66% under the WS, WM, and WF rotation treatments, respectively. The mean soil water content in 2011–2012 were 13.79%, 13.00%, and 13.39% under the straw retention treatments TS, HS, and NS, respectively. During the winter wheat growing season of 2012–2013, out of the 13 monitoring missions that were conducted, we found highly significant and significant differences in the soil water content test between the three rotation treatments five times and three times, respectively (both P > 0.05, Table 1), and there were significant differences between the three straw retention treatments twice (Table 1). The mean soil water content in 2012–2013 were 14.62%, 14.90%, and 15.27% under the WS, WM, and WF rotation treatments, respectively. The mean soil water content in 2012–2013 were 15.01%, 14.79%, and 15.00% under the straw retention treatments TS, HS, and NS, respectively.

### Soil organic carbon

**Fig 5:**
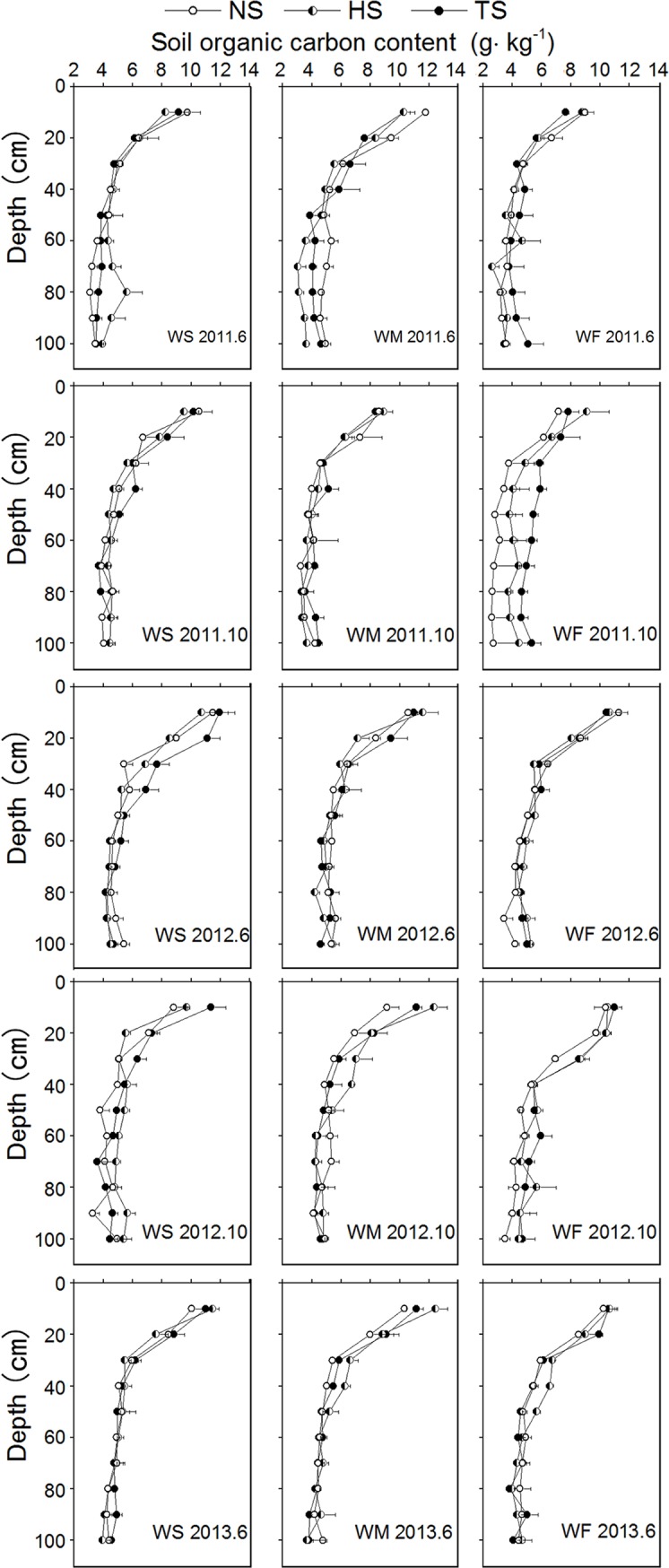
Effect of rotation mode and straw retention amounts on soil orgnic carbon content.

The soil organic carbon concentration was the highest in the 0–10 cm soil layer, and decreased with increasing soil depth (Fig 5). The organic carbon concentration was below 10 g/kg of soil at depths below 10 cm. Under the same rotation treatment, different soil layers had the same pattern of organic carbon concentration. There were differences in soil organic carbon concentrations under the three rotation treatments. After four years of different straw management and plant rotation treatments, there were differences in SOC concentrations between the different treatments. Soil organic carbon concentrations had a "W" type change, i.e., SOC concentrations increased after the winter wheat harvest, but decreased during summer crop growth period, indicating that growing winter wheat enhanced the function of soil as a carbon pool. The mixed-model analysis indicated highly significant differences in the SOC of the 0–10 cm and 11–40 cm soil depth layers at the period of 2011–6, and significant differences between the three rotation treatments (Table 2). Additionally, there was a significant difference in the SOC of the 11-40cm soil depth layer at the period of 2011–10 between the three straw treatments. The mean organic carbon concentrations of soils (0–100 cm, June 2011 to June 2013) under the WS rotation were 6.91, 6.63, and 6.39 g/kg (for TS, HS, and NS treatments), for the WM rotation the mean SOC concentrations were 6.90, 6.72, and 6.57 g/kg (for TS, HS, and NS treatments), and for the WF rotation the mean SOC concentrations were 6.49, 6.52, and 6.37 g/kg (for TS, HS, and NS treatments).

**Table 2:** Effect of crop rotation and straw retention mounts on SOC during the period from 2011 to 2013. (**P<0.01).

Time	Source	0-10cm		11-40cm		41-100cm	
		F Value	Significance	F Value	Significance	F Value	Significance
2011–5	rotation	10.546	0.001 **	10.005	0.000 **	0.314	0.747
	straw	3.020	0.069	0.326	0.723	0.040	0.961
2011–10	rotation	5.794	0.010 *	4.695	0.011 *	0.667	0.562
	straw	0.506	0.610	3.448	0.035 *	1.752	0.284
2012–6	rotation	0.394	0.679	1.272	0.286	1.635	0.303
	straw	0.030	0.970	2.543	0.085	0.001	0.999
2012–10	rotation	0.631	0.578	15.191	0.000 **	0.083	0.922
	straw	2.566	0.192	2.297	0.105	1.195	0.392
2013–6	rotation	1.471	0.251	0.860	0.427	1.690	0.188
	straw	3.222	0.059	1.013	0.368	0.204	0.816

**Fig 6:**
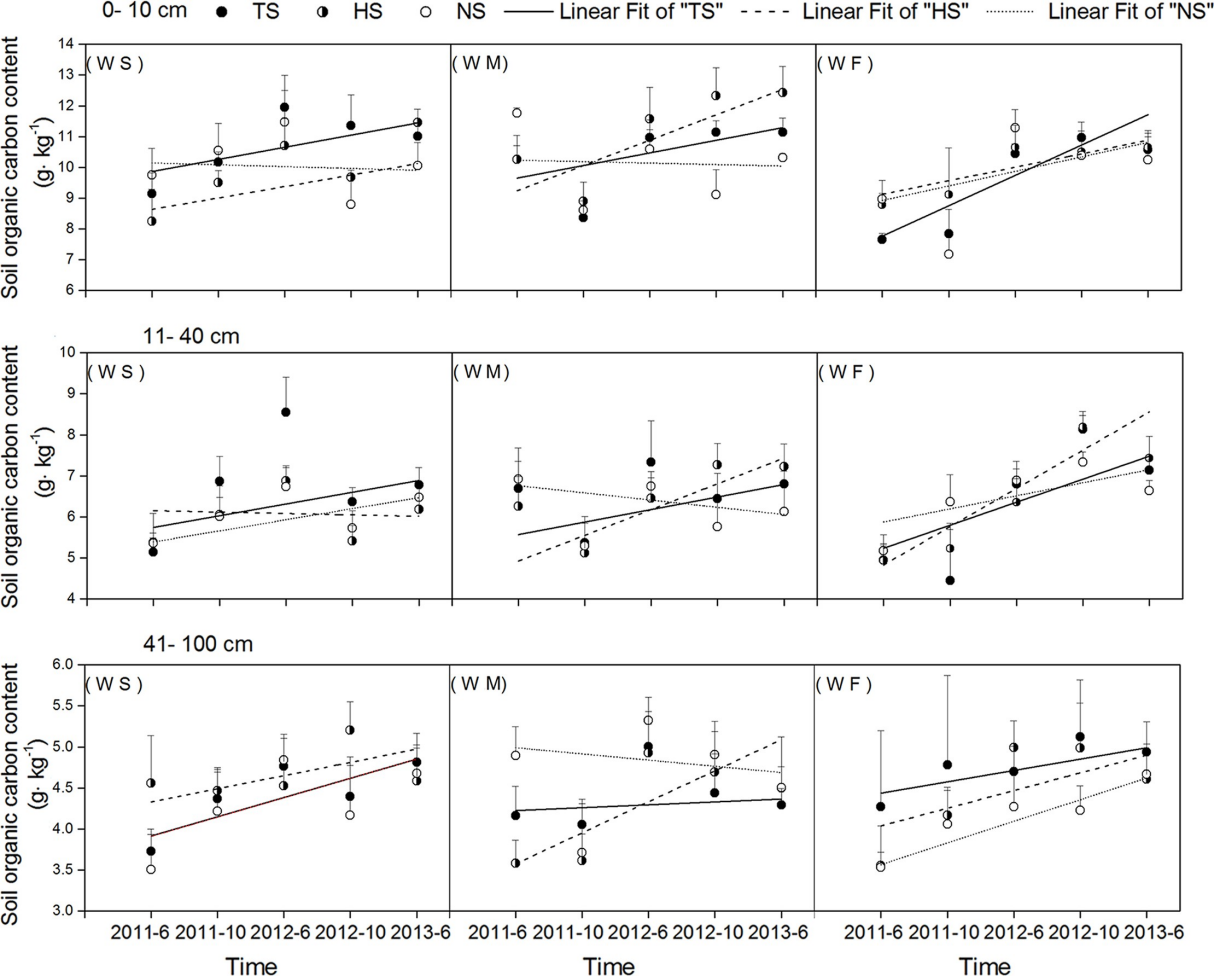
Mean soil organic carbon content of 0-100cm of three straw treatments and three rotation treatments.

The results indicate that mean soil organic carbon concentrations in the period from June 2011 to June 2013 increased with the amount of straw retention, and all treatments of the three rotation modes showed similar patterns (Fig 6). The mean organic carbon concentration of soils at 0–10 cm depth under the different rotations were as follows: WS rotation: 10.72, 9.92, and 10.12 g/kg, for TS, HS, and NS treatments; WM rotation: 10.37, 11.09, and 10.08 g/kg, for TS, HS, and NS treatments; WF rotation: 9.50, 9.94, and 9.61 g/kg, for TS, HS, and NS treatments. The mean soil organic concentrations at 11–40 cm soil depth under the different rotations were as follows: WS rotation: 6.74, 5.98, and 6.06 g/kg, for TS, HS, and NS treatments: WM rotation: 6.53, 6.47, and 6.17 g/kg, for TS, HS, and NS treatments; WF rotation: 6.30, 6.43, and 6.48 g/kg, for TS, HS, and NS treatments. The mean soil organic carbon concentrations at 41–100 cm of soil depth were as follows: WS rotation 4.41, 4.67, and 4.28 g/kg for TS, HS, and NS treatments; WM rotation: 4.39, 4.26, and 4.67 g/kg for TS, HS, and NS treatments; WF rotation: 4.66, 4.44, and 4.15 g/kg, for TS, HS, and NS treatments.

### Correlations between environmental factors and soil respiration

**Fig 7:**
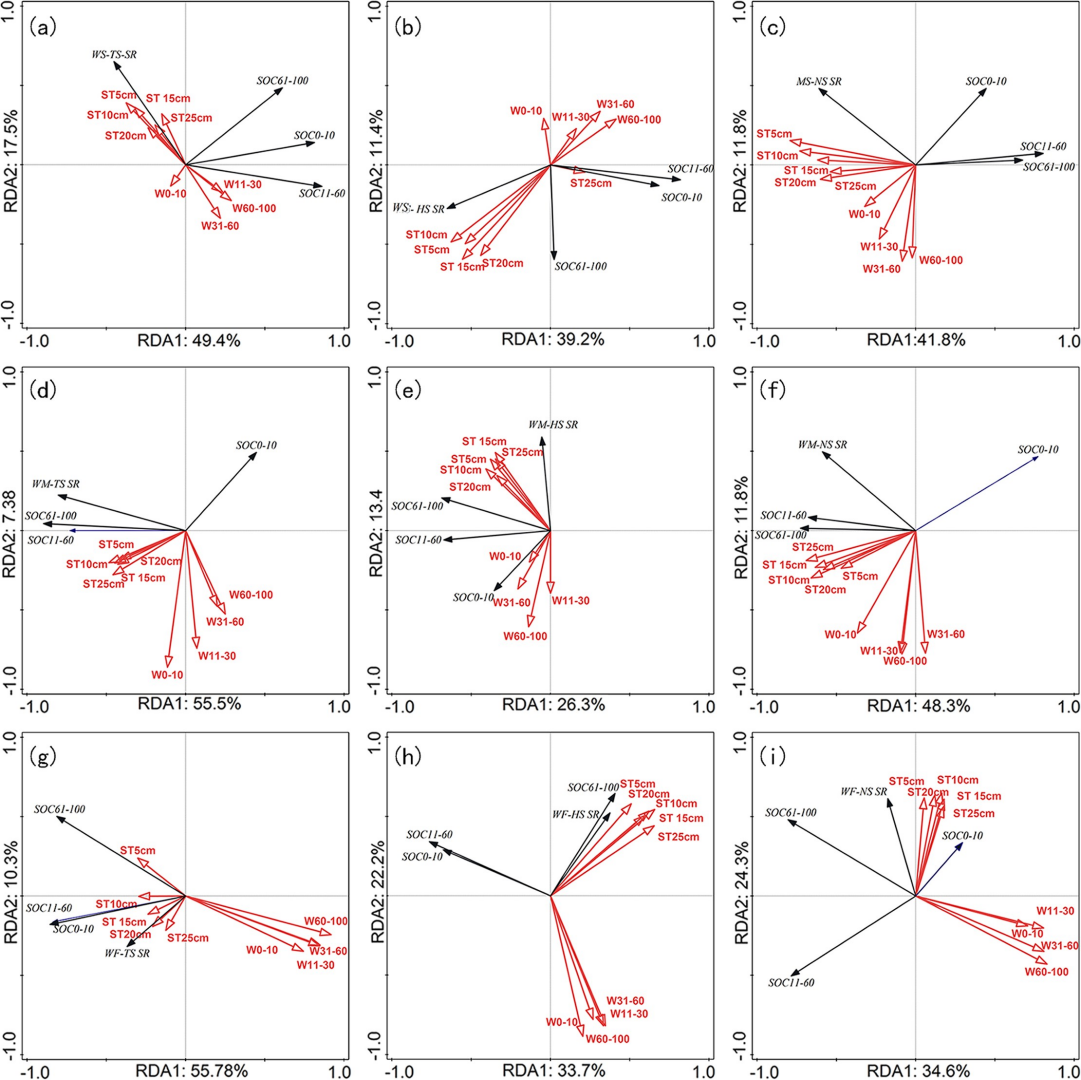
Ordination plots of the results from the redundancy analysis (RDA) to identify the relationships among the soil respiration (SR), soil origin carbon (SOC) (Black arrows) and the temperature and soil water content (Red arrows). **ST, W is the soil temperature and soil water content of different depth.** (a- i) is the relationships between different treatments of rotation and amount of straw retention. (a): WS Rotation plus TS, (b): WS Rotation plus HS; (c): WS plus NS; (d): WM Rotation plus TS;(e):WM Rotation plus HS; (f): WM Rotation plus NS; (g): WF Rotation plus TS; (h): WF Rotation plus HS; (i): WF Rotation plus NS.

Redundancy analysis (RDA) was used to determine the relationships between soil temperature and soil respiration (Fig 7). The results indicate that the soil respiration rate was strongly correlated with soil temperature and soil organic carbon concentrations under the nine treatments of three straw retention levels and three rotation treatments during the two year study. Temperature was the main determinant of soil respiration rates due to its influence on the kinetics of microbial decomposition, root respiration, and the diffusion of enzymes and substrate. Our results suggest that the relationship between soil respiration and temperature was strongest at a depth of 10 cm. Correlation analysis showed that the nine treatments affecting soil water content were negatively correlated with soil respiration rate. Soil water content can decrease soil porosity, which in turn reduces the outward release of CO_2_ from deep soil.

## Discussion

### Effect of crop rotation treatment on soil respiration, soil water content and temperature

Throughout the two years of crop growing seasons, we found highly significant and significant differences in the soil respiration test between the three rotation treatments six and five times, respectively. Our data indicate that mean soil respiration under the WS rotation treatment were lower than from soils under the WM and WF rotation treatments during the growing seasons of two years. Due to differences in land use, tillage, cropping practices, soil fertilization and precipitation, the mean soil respiration recorded in this study were more than 10 times larger than those recorded from the Taklimakan Desert [4]. These results demonstrate that the mean soil organic carbon concentration of the soil (0–100 cm depth), from June 2011 to June 2013, was higher for WM than for the other rotation treatments. When the NS treatment was combined with the WM and WM rotation treatments, the SOC concentration in the topsoil (0–10 cm and 11–40 cm) had a decreasing trend after two years of planting, but when the NS treatment was combined with the WF rotation treatment, the SOC concentration in the topsoil had an increasing trend. Our study also showed that growing winter wheat enhanced the function of soil as a carbon pool, but summer crops consumed the soil organic carbon, this result was consistent with the finding by Lai [9]. Compared with maize, soybeans, a legume crop, resulted in lower rates of soil respiration, and the ability of soybeans to fix atmospheric nitrogen symbiotically reduces the need for nitrogen fertilizer application. As such, planting soybeans enables farmers to save energy and reduce greenhouse gas emissions, and this result is in accordance with the results of most previous studies [37].

Throughout the two years of crop growing seasons, due to the potential evaporation under three crop rotation is differences [30], we found highly significant and significant differences in the soil water content test between the three rotation treatments 13 and four times, respectively. Our results clearly demonstrate that the WF rotation resulted in a lower soil temperature and a higher percentage of soil water content. Because no crops are planted during the summer of the WF rotation, the consumption of precipitation by plants is low. Our experiment also demonstrated that soil water content under different rotation treatments was affected by rainfall, and there were large changes in soil water content during the growth period of the different crops, similar dynamic were reported by Yang [4].

### Effects of straw retention on the soil respiration, soil water content, and soil temperature

In north-west China, crop residues are commonly burned for cooking and heating [13]. However, this residue management practice is not environmentally friendly, and may not be sustainable. Previous studies have shown that straw residue management practices may lead to significant changes in the soil physical, chemical, biological and biochemical properties [20], and thereby affect the composition, distribution, and activities of the soil microbial communities which may increase the crop yield and enzyme production [38]. Older studies mostly used single straw types, such as maize, wheat, or soybean stalks in their experiment. However, this study evaluated three crop rotation treatments, so, wheat straw, maize straw, and soybean straw were used.

Throughout the two years of crop growing seasons, we found highly significant and significant differences in the soil respiration test between the three straw retention treatments one and two times, respectively. Previous studies demonstrated that straw incorporation may increase soil respiration [20, 39], but our results suggest that the mean soil respiration rates under the TS (5.88 μmol·m^-2^·s^-1^), and HS (5.82 μmol·m^-2^·s^-1^) treatments were lower than that of soils under the NS (6.14 μmol·m^-2^·s^-1^) treatment, this is not consistent with what has been found in previous studies [20]. There are two possible reasons for this: the first is that soil respiration were positively correlated with the concentration of dissolved organic C (DOC) [31], and that the incorporation of residue plus urea reduced the concentration of DOC [40]. Ren reported that microbial respiration can be limited by access to carbon substrates, and can decrease the diffusion of carbon substrates [31]. Another possible reason is that decomposition rates for both soybean and maize residues placed on the soil surface were higher than those of the same residues incorporated into the soils [21]. In this experiment, both soybean and maize straw residues were distributed in the soil by rotary tillage at a depth of 0–10 cm, and winter wheat was sown before the straw was completely decomposed in the field. The undecomposed straw may increase the total soil porosity and capillary porosity, resulting in lower soil temperatures and a high rate of soil water loss compared to the NS treatment in winter. This may create unfavorable conditions for winter wheat growth, and result in yield reduction, as was also found by Chen (2013) [41]. This also explains why the mean soil temperatures of soils under the NS treatment were higher than in soils under the TS and HS treatments during the crop growth period. A similar conclusion was reached by Mahdi (2005), who reported that soil respiration from plots which contained straw retention were 24% lower than those of soils from plots which did not contain straw retention [42].

Changes in soil water content have a different effect on the soil microbial activity and on the activity of microbial decomposers of straw retention [43]. Some studies have shown that lower rainfall conditions significantly reduced the soil total microbial biomass, bacterial abundance, and soil organic carbon and soil respiration [44]. In this study, the mean soil respiration of the 2011–2012 winter wheat growth period were higher than the mean soil respiration of the 2012–2013 winter wheat period, perhaps due to the fact that soil water content was lower during the 2011–2012 winter wheat growth period than during the 2012–2013 winter wheat growth period. Soil respiration tended to decrease with increasing soil water filled pore space, similar conclusion was reached by Wang (2014) [45]. The mean soil water content of soil at 0–100 cm depth under TS treatments was higher than of soils under the HS and NS treatments, and this suggests that the incorporation rate of straw can enhance precipitation water use efficiency, as was also reported by Wang [46]. However, there were no statistically significant differences in soil water content under the different straw retention treatments.

Straw return may improve soil nutrient availability, which may favor crop growth, which can in turn increase ecosystem carbon input [20]. The results of this experiment show that during the crop growth period, straw retention can increase the soil organic carbon concentration during the growth period, and the soil organic carbon concentration increased with increased straw retention. The TS and HS treatments resulted in soil organic carbon concentrations that were by 8.14% and 3.76% higher than under the NS treatment under the WS rotation, 5.02% and 2.28% higher than under the NS treatment under the WM rotation, and 1.88% and 2.35% higher than under the NS treatment under the WF rotation. However, the differences between soil SOC under the TS and HS and NS treatments found in our study are lower than those reported (by 12%) in previous studies [47, 48]. This may be due to the relatively short duration of this experiment.

### Effects of the crop rotation and straw retention on soil respiration, soil water content, and temperature

We found that crop rotation and straw retention management treatments influenced the soil CO_2_ respiration rate. After the summer crop was harvested, soybean and maize straw was retained in the field. Our results demonstrate that, during the winter wheat growth period of the WS rotation treatment, the soil respiration rate under the TS straw retention treatment was greater than under the HS treatment. However, under the WM rotation treatment, the respiration rate of soils under the TS straw retention treatment was lower than that of soils under the HS treatment. The same trend was found during both years of the experiment. A possible reason for this is that soybean straw has a lower C/N ratio, and maize straw has a higher C/N ratio [49]. The decomposition rate of straw depends on the C/N ratio, and straw with a lower C/N ratio decomposes faster than straw with a higher C/N ratio [50].

For decades, crop retention as a cultivation management measure was widely thought to be an effective way to improve SOC accumulation, and soil quality [20]. This study demonstrates that soil organic carbon in topsoil (0–10 cm and 11–40 cm) was decreased trend under the NS treatment under WM and WS rotation treatments, but it was increased trend under the NS treatment under the WF rotation treatment during the two years experiment. Compared to soils under the NS treatment, soils under the TS and HS treatments had a higher carbon input and lower soil respiration rate, and straw retention practices have the potential to improve carbon sequestration. These findings are directly in line with previous findings [47]. As such, straw incorporation practices should be encouraged in north-west China.

Soil temperature and soil water content affect the soil respiration and uptake of gases through their effects on microorganisms and root activities [8]. Results of this study indicate that, during the growth period, soil under the NS treatment had a higher mean soil respiration rate compared to soil under the TS and HS treatments. This is probably because soil porosity tended to increase due to straw incorporation, and when straw is not fully decomposed at the beginning of the next crop growth period, this may increase the speed of soil water evapotranspiration, and result in lower soil temperatures in the winter. This ultimately strengthened the relationship between the soil respiration and temperature. Soil temperature, particularly at a depth of 10 cm, can explain more of the variation in soil respiration than soil water content parameters, and this is line with previous findings of Kristofor [15].

## Conclusions

This study demonstrates that soil respiration, temperature, and water content in the field is mainly affected by the practice of crop rotation. On this basis, we conclude that WS rotation resulted in lower rates of soil respiration rate, WM rotation resulted in a higher mean soil organic carbon concentration, and WF rotation resulted in lower soil temperature and higher soil water content percentages. Correlation analysis indicated that soil respiration had a strong relationship with soil temperature, and did not increase as the straw retention amount increased. Our results suggest that the mean soil respiration rates of soils under TS and HS treatments were lower than that of soils under the NS treatment. This study demonstrated that soil organic carbon concentrations increased during the winter wheat growth period, but decreased during the summer crop growth period. SOC in topsoil had decreased trend under NS treatment under the WM, and WS rotation treatments, but it had increased trend under the NS treatment of WF rotation treatment during the two year experiment. Compared to soils under the NS treatment, soils under the TS and HS treatments had a higher carbon input and lower soil respiration rate, and straw retention practices have the potential to improve soil carbon sequestration.
